# Enhanced ε-Poly-L-Lysine Production by the Synergistic Effect of ε-Poly-L-Lysine Synthetase Overexpression and Citrate in *Streptomyces albulus*

**DOI:** 10.3389/fbioe.2020.00288

**Published:** 2020-04-22

**Authors:** Aixia Wang, Wenzhe Tian, Lei Cheng, Youqiang Xu, Xiuwen Wang, Jiayang Qin, Bo Yu

**Affiliations:** ^1^College of Pharmacy, Binzhou Medical University, Yantai, China; ^2^Beijing Engineering and Technology Research Center of Food Additives, Beijing Technology and Business University (BTBU), Beijing, China; ^3^CAS Key Laboratory of Microbial Physiological and Metabolic Engineering, Institute of Microbiology, Chinese Academy of Sciences, Beijing, China

**Keywords:** *Streptomyces albulus*, ε-PL synthase, overexpression, citrate, synergistic effect

## Abstract

ε-Poly-L-lysine (ε-PL) is a natural amino acid polymer produced by microbial fermentation. It has been mainly used as a preservative in the food and cosmetics industries, as a drug carrier in medicines, and as a gene carrier in gene therapy. ε-PL synthase is the key enzyme responsible for the polymerization of L-lysine to form ε-PL. In this study, the ε-PL synthase gene was overexpressed in *Streptomyces albulus* CICC 11022 by using the *kasO*p^∗^ promoter and the ribosome binding site from the capsid protein of phage ϕC31, which resulted in a genetically engineered strain Q-PL2. The titers of ε-PL produced by Q-PL2 were 88.2% ± 8.3% higher than that produced by the wild strain in shake flask fermentation. With the synergistic effect of 2 g/L sodium citrate, the titers of ε-PL produced by Q-PL2 were 211.2% ± 17.4% higher than that produced by the wild strain. In fed-batch fermentations, 20.1 ± 1.3 g/L of ε-PL was produced by *S. albulus* Q-PL2 in 72 h with a productivity of 6.7 ± 0.4 g/L/day, which was 3.2 ± 0.3-fold of that produced by the wild strain. These results indicate that ε-PL synthase is one of the rate-limiting enzymes in ε-PL synthesis pathway and lays a foundation for further improving the ε-PL production ability of *S. albulus* by metabolic engineering.

## Introduction

ε-Poly-L-lysine (ε-PL) is a kind of polymer composed of 25–35 L-lysine residues connected by α-amino group and ε-carboxyl group to form an amide bond. It was first separated and purified by Japanese scholars Shima and Sakai in the fermentation broth of *Streptomyces* (Shima and Sakai, 1977). ε-PL has many excellent properties, including that it is antibacterial, biodegradable, water soluble, thermostable, edible, and non-toxic. Therefore, ε-PL has been used as a preservative in food, cosmetics, health care, and other industries (Xu et al., 2016). In addition, ε-PL can also be used as gene carrier, drug carrier, in weight loss and health care products, as a new water absorbent material, biochip, and bioelectronic coating agent (Xu et al., 2016).

ε-Poly-L-lysine is produced mainly by microbial fermentation. It was proven that L-lysine is the precursor of ε-PL synthesis by using isotope technology (Shima et al., 1983). Kawai et al. (2003) reported the synthesis of ε-PL by using a cell-free system. They found that the synthesis activity was on the cell membrane, and the enzyme activity depended on ATP and was not affected by ribonuclease, kanamycin, or chloramphenicol. These results indicate that ε-PL synthesis is a membrane protein-catalyzed reaction. Five years later, the ε-PL synthase (Pls) was purified from *Streptomyces albulus* NBRC 14147 (Yamanaka et al., 2008). It was found that the enzyme has a molecular weight of 130 kDa and is an unusual non-ribosomal peptide synthetase with adenylation. In addition, the thiolation domain, without the condensation or thioesterase domain of a traditional peptide synthetase, has six transmembrane domains surrounding three tandem soluble domains, which use free L-lysine polymers (or the monomer in the initial reaction) as a receptor and Pls-bound L-lysine as a donor to continuously catalyze the polymerization of L-lysine, directly producing chains of different lengths. After that, Yamanaka et al. (2011) reported a recombinant Pls expression system that can be used for site-specific mutation analysis. They attempted to express Pls in *S. albulus* using the constitutive promoter *ermE*^∗^ but were unsuccessful. Therefore, they speculated that the expression of Pls gene (*pls*) requires its own promoter, which is likely regulated in secondary metabolism. The *pls* gene from *S. albulus* was heterologously expressed in *S. lividans*, and the recombinant strain was capable of synthesizing ε-PL. The own promoter of *pls* is still used in their study (Geng et al., 2014). Recently, Xu et al. (2019) found a strain capable of producing short-chain ε-PL, *Kitasatospora aureofaciens*, and they attempted to heterologously express the *plsII* gene of this strain in *S. albulus*. They tested three promoters, namely, the constitutive promoter *ermE*^∗^, the *plsII* gene’s own promoter from *K. aureofaciens*, and the *plsI* gene’s own promoter from *S. albulus*. The results showed that *ermE*^∗^ could not start the expression of *plsII* gene, whereas the other two promoters could. The final ε-PL concentration obtained by the *plsI* gene’s own promoter from *S. albulus* was 34.1% higher than that by the *plsII* gene’s own promoter from *K. aureofaciens*.

The above results indicate that there is no precedent for the successful expression of *pls* gene using other promoters, except the gene’s own promoter. In this study, an engineered strong promoter *kasO*p^∗^ (Wang et al., 2013) and the ribosome binding site (RBS) from the capsid protein of phage ϕC31 (Smith et al., 1999; Bai et al., 2015) were combined to overexpress the *pls* gene in *S. albulus*, and the ability of the gene-engineered strain to produce ε-PL was also investigated.

## Materials and Methods

### Strains and Plasmids

The strains and plasmids used in this study are shown in Table 1. The ε-PL production strain *S. albulus* CICC 11022 was purchased from the China Industrial Microbial Culture Collection (CICC). *Escherichia coli* ET12567/pUZ8002 (Paget et al., 1999) was used as the non-methylating plasmid donor strain for intergeneric conjugation with *S. albulus* CICC 11022. The *E. coli*/*S. albulus* shuttle vector, pSET152, which can integrate specifically into the *attB* sites on the *Streptomyces* chromosome via integrase-attp-directed site-specific recombination, was used for *pls* gene overexpression.

**TABLE 1:** Strains and plasmids used in this study.

Strain and plasmid	Relevant genotype^a^	Source or references
**Strains**		
*Streptomyces albulus* CICC 11022	Control strain, transformation host	CICC
*S. albulus* Q-PL2	Overexpression strain, *S. albulus* CICC 11022 harboring pSET152-pro-rbs2-pls	This study
*Escherichia coli* ET12567/pUZ8002	*rec*E, *dcm*^–^, *dam*^–^, *hsd*S, Cm^r^, Tet^r^, Str^r^, Km^r^	Paget et al., 1999
*E. coli* Top10	F^–^*mcr*A Δ(mrr-hsdRMS-mcrBC) φ80*lac*ZΔM15 Δ*lac*X74 *rec*A1 *ara*D139 Δ(*ara*-*leu*)7697 *gal*U *gal*K *rps*L (Str^R^) *end*A1 *nup*G	Invitrogen
Plasmids		
pSET152	5.7 kb, Apr^r^, integrative plasmid, *lacZ*α *ori*^pUC19^*oriT*^RP4^*int-attP^φ^* ^31^*aac*(*3*)*IV*	Bierman et al., 1992
pSET152-pro-rbs2-pls	Apr^r^, pSET152 carrying *kasO*p* promoter, RBS2, and *pls* gene	This study

*^*a*^Cm, chloramphenicol; Tet, tetracycline; Str, streptomycin; Km, kanamycin; Apr, apramycin.*

### Strain Culture and Fermentation Conditions

*Escherichia coli* was cultured under aerobic condition at 37°C using Luria–Bertani (LB) medium, which contained 10 g/L tryptone, 5 g/L yeast extract, and 10 g/L sodium chloride. *S. albulus* was cultured at 30°C. The medium used to culture the spores of *S. albulus* was MS solid medium, which contains 20 g/L of mannitol, 20 g/L of soybean powder, and 20 g/L of agar powder. M3G medium was used for the seed culture of *S. albulus*, which is composed of 50 g/L of glucose, 10 g/L of (NH_4_)_2_SO_4_, 5 g/L of yeast extract, 0.5 g/L of MgSO_4_⋅7H_2_O, 0.8 g/L of K_2_HPO_4_, 1.36 g/L of KH_2_PO_4_, 0.03 g/L of FeSO_4_⋅7H_2_O, 0.04 g/L of ZnSO_4_⋅7H_2_O and has an initial pH of 6.8. For shake flask fermentations of *S. albulus*, M3G medium was used as the fermentation medium after appropriate modification according to the experimental requirements. For fed-batch fermentations of *S. albulus*, 25 g/L glucose and 25 g/L glycerin were used as the mixed carbon source, 5 g/L sodium citrate was added, and the other components were the same as M3G medium without glucose. When required, antibiotics were used at the following concentrations: 50–80 μg/mL apramycin, 25–50 μg/mL chloramphenicol, 40–50 μg/mL kanamycin, and 25 μg/mL nalidixic acid.

In the shake flask fermentations of ε-PL, the *S. albulus* strains were streaked onto MS solid medium, and spores were collected after 5–6 days of cultivation at 30°C. Then, 400-μL spore solution was inoculated into 300-mL conical flask containing 50 mL seed medium, and seed culture was obtained after 48 h of shaking cultivation at 30°C and 220 rpm unless otherwise specified. Then, the seed culture was inoculated into conical flasks containing 50 mL of fermentation medium at 10% (volume ratio). After 72 h of cultivation at 30°C and 220 rpm, the concentration of ε-PL and other parameters in the fermentation broth were determined.

Fed-batch fermentations of ε-PL by *S. albulus* were conducted in a 2-L bioreactor (NBS, Germany) containing 1 L of fermentation medium. The preparation of the seed culture was the same as that in shake flask fermentation. The seed culture was inoculated into the fermenter at 10% volume ratio, and the temperature was controlled at 30°C. The aeration was 3 vvm, and the dissolved oxygen level was controlled at approximately 30% by adjusting the stirring speed at 200–800 rpm. According to Ren et al. (2015), the pH was not controlled in the early stage of fermentation. When the pH of the fermentation broth dropped to 4.0, ammonium hydroxide was added to maintain the pH at 4.0. Feeding medium was added to the fermentation broth when the glucose concentration in the fermentation broth was less than 10 g/L. The composition of the feeding medium was 250 g/L of glucose, 250 g/L of glycerin, 100 g/L of ammonium sulfate, and 50 g/L of sodium citrate. Cell growth, substrate consumption, and ε-PL production were measured every few hours. Three separate experiments were conducted for all the shake flask and fed-batch fermentations.

### Molecular Manipulations

Genomic DNA was extracted using the TIANamp Bacteria DNA Kit (TIANGEN, China). Phanta Super-Fidelity DNA Polymerase was used to amplify the *pls* gene (Vazyme, China). Plasmid DNA was isolated using the *Easypure*^®^ Plasmid Miniprep Kit (Transgen, China). The *EasyPure*^®^ Quick Gel Extraction Kit (Transgen, China) was used for DNA purification. Oligonucleotides were prepared by Invitrogen (Shanghai, China). The Gibson Assembly^®^ Cloning Kit (NEB, England) was used to integrate the vector and the DNA inserts.

### Construction of *pls* Gene Overexpressing Strain

The oligonucleotides used to construct *pls* gene overexpression plasmids are shown in Supplementary Table S1. The ligation product *pro-rbs2* of the strong promoter *kasO*p^∗^ and the ribosome-binding site (RBS2) from the capsid protein of phage ϕC31 were obtained by annealing oligonucleotides pro1, pro2, and pro4. The primers pls-F2 and pls-R were used to amplify the *pls* gene from the genome of *S. albulus* CICC 11022. The PCR conditions were as follows: 94°C for 30 s, 55°C for 30 s, and 72°C for 2 min, for 30 repeated cycles each. The DNA fragments of *pro-rbs2* and *pls* gene were mixed as the template to perform overlapping PCR using primers pro1 and pls-R, and a complete expression element, namely, *pro-rbs2-pls—*containing the strong promoter *kasO*p^∗^, RBS2, and *pls* gene—was finally obtained. The PCR conditions wer as follows: 94°C for 30 s, 55°C for 30 s, and 72°C for 2 min, for 30 repeated cycles each. The expression element *pro-rbs2-pls* was ligated with *Xba*I and *Eco*RI double-digested vector pSET152 by Gibson reaction and transformed into *E. coli* Top10. The correctly ligated transformants were screened and verified by sequencing. The sequence of *pls* gene in *S. albulus* CICC 11022 shows 100% identity to the *pls* gene in *S. albulus* PD-1 (accession no. JF427577). The recombinant overexpression plasmid was named pSET152-pro-rbs2-pls (Supplementary Figure S1), and the sequence of *pro-rbs2-pls* is shown in Supplementary Figure S2.

The constructed *pls* gene overexpression plasmids pSET152-pro-rbs2-pls were first transferred into *E. coli* ET12567/pUZ8002 and then transferred into *S. albulus* CICC 11022 by intergeneric conjugation, according to a previously reported method (Xu et al., 2015) with some modifications. The specific steps were as follows.

A single colony of *E. coli* ET12567/pUZ8002 harboring pSET152-pro-rbs2-pls was selected from LB solid medium containing 50 μg/mL kanamycin, 50 μg/mL chloramphenicol, and 50 μg/mL apramycin. The cells were collected at 37°C at an OD_600_ of 0.6, washed three times with fresh LB medium, and finally resuspended in 200 μL LB for later use. Meanwhile, the spores of *S. albulus* CICC 11022 were suspended in 400 μL of 2 × YT medium, which contains 16 g/L tryptone, 10 g/L yeast extract, and 5 g/L sodium chloride. After a 10 min heat shock at 50°C, the spores were cooled to room temperature, mixed with the prepared donor strain, and cultivated at 30°C for 1 h with shaking at 100 rpm/min. The mixed bacterial solutions were plated onto MS solid medium. After 14 h, the plates were covered with 1 mL of sterile water containing 80 μg/mL apramycin and 25 μg/mL nalidixic acid. The plates were further incubated at 30°C for approximately 2 days, and the ex-conjugants were obtained. The spores were cultured to obtain the genetically engineered strain *S. albulus* Q-PL2, which harbor pSET152-pro-rbs2-pls.

### Analytical Method

ε-Poly-L-lysine concentration was determined using the method described previously (Pan et al., 2017). Glucose concentration was measured using an SBA-40E biosensor analyzer (Shangdong Academy of Sciences, China) (Yin et al., 2019). The concentration of glycerol in the culture supernatant was measured using a Glycerol GK Assay Kit (Megazyme, Ireland) (Lenzen et al., 2019). Cell growth was measured by detecting the optical density (OD) of the samples at 600 nm in a spectrophotometer. All samples were measured three times. All the figures were plotted using GraphPad Prism 7 software (GraphPad Software, United States). Statistical analysis was performed using unpaired *t* test or one-way ANOVA with Dunnett’s multiple comparisons test or Sidak’s multiple comparisons test in GraphPad Prism.

### Comparison of *pls* Gene Expression by Quantitative Real-Time PCR

Quantitative Real-Time PCR was used to compare the expression levels of *pls* gene in different *S. albulus* strains. Spores of the wild strain CICC 11022 and the genetically engineered strain Q-PL2 were collected from MS solid medium, inoculated into M3G medium, and cultured at 30°C with shaking for 60 h. Samples were collected every 12 h for RNA isolation. Total RNA was extracted with an *EasyPure*^®^ RNA Kit (Transgen, China). The cDNA was reverse transcribed using *EasyScript*^®^ One-Step gDNA Removal and cDNA Synthesis SuperMix (Transgen, China), and qRT-PCR was performed using a QuantiNova SYBR Green PCR kit (Qiagen, Germany) on a LightCycler 96 instrument (Roche, Germany). The PCR conditions were as follows: 95°C for 5 s and 60°C for 10 s, for 40 repeated cycles. The RNA polymerase sigma factor (hrdB) was selected as the reference gene (Xu et al., 2018). The genes and primers used for qRT-PCR are shown in Supplementary Table S2. The relative gene expression data were analyzed using the 2^–ΔΔ*Ct*^ method, as described by Qin et al. (2015). All qRT-PCR runs were conducted with three biological and three technical replicates.

## Results

### Comparison of *pls* Gene Expression Levels in Wild and Genetically Engineered *S. albulus*

**FIGURE 1:**
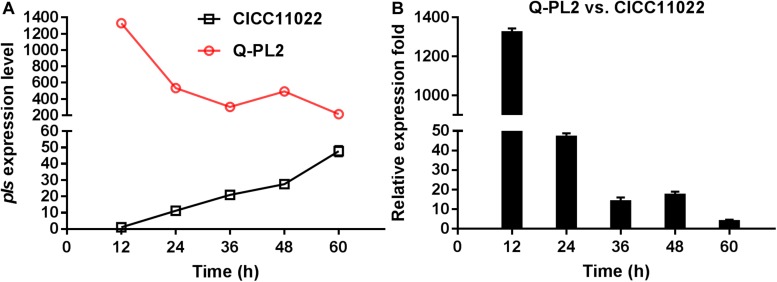
Comparison of *pls* gene expression by qRT-PCR. **(A)** Expression level of *pls* gene in CICC 11022 and Q-PL2 during fermentation. **(B)** Relative expression fold of *pls* gene between Q-PL2 and CICC 11022 at different fermentation times. Data represent the means of three separate experiments, and error bars represent the standard deviation. Some error bars cannot be seen due to small standard deviations.

The expression level of the *pls* gene at 12 h in *S. albulus* CICC 11022 was defined as 1, and *hrdB* was used as the internal reference gene. The relative quantitative method was used to calculate the expression levels of the *pls* gene at 24, 36, 48, and 60 h in strain CICC 11022 and those of *pls* genes at 12, 24, 36, 48, and 60 h in genetically engineered strain Q-PL2. The results are shown in Figure 1A. In the wild strain CICC 11022, the expression of *pls* gene increased slowly with the increase of culture time, reached the maximum value at 60 h, and had an increasing trend. The results of Yamanaka et al. (2010) showed that the *pls* gene is transcriptionally regulated in the mid-log and stationary phases of strain growth, which is consistent with our results of strain CICC 11022. The constitutive promoter *kasO*p^∗^ was used in the genetically engineered strain Q-PL2. The expression levels of the *pls* gene in Q-PL2 reached the maximum at the beginning and then gradually decreased, but they were always much higher than those in CICC 11022. Figure 1B shows the relative expression folds of *pls* gene between Q-PL2 and CICC 11022 at different time points. These results indicate that the constitutive promoter *kasO*p^∗^ together with RBS2 can significantly improve the expression level of the *pls* gene, especially in the early stage of fermentation.

### Effect of *pls* Gene Expression on ε-PL Production by *S. albulus*

**FIGURE 2:**
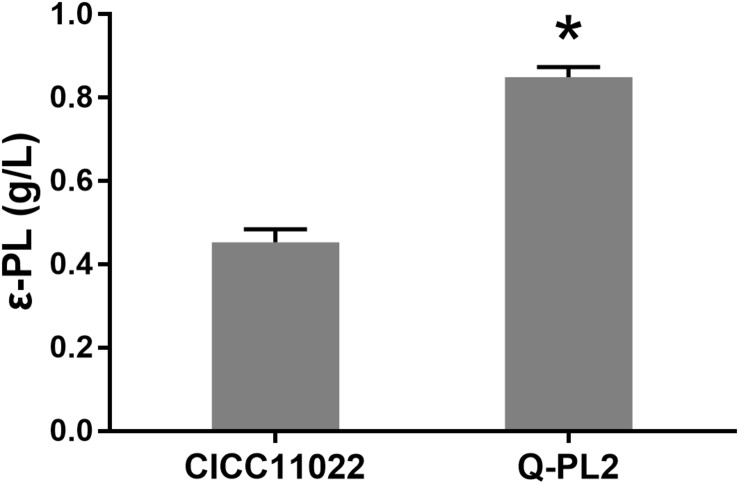
Effect of *pls* gene expression on ε-PL production by *S. albulus*. Data represent the means of three separate experiments, and error bars represent the standard deviation. Some error bars cannot be seen due to small standard deviations. *p < 0.05; unpaired *t* test.

The ε-PL production ability of *S. albulus* CICC 11022 and the *pls* gene overexpressing strain Q-PL2 was compared using M3G medium. The results are shown in Figure 2. After 72 h of fermentation, the titers of ε-PL produced by CICC 11022 and Q-PL2 were 0.45 ± 0.03 and 0.85 ± 0.02 g/L, respectively. The latter was 88.2 ± 8.3% higher than the former. The results confirm that *pls* gene overexpression can improve ε-PL production of *S. albulus*. However, the increase of ε-PL production did not match the increase of *pls* gene overexpression.

### Synergistic Effect of *pls* Gene Overexpression and Citrate on ε-PL Production

**FIGURE 3:**
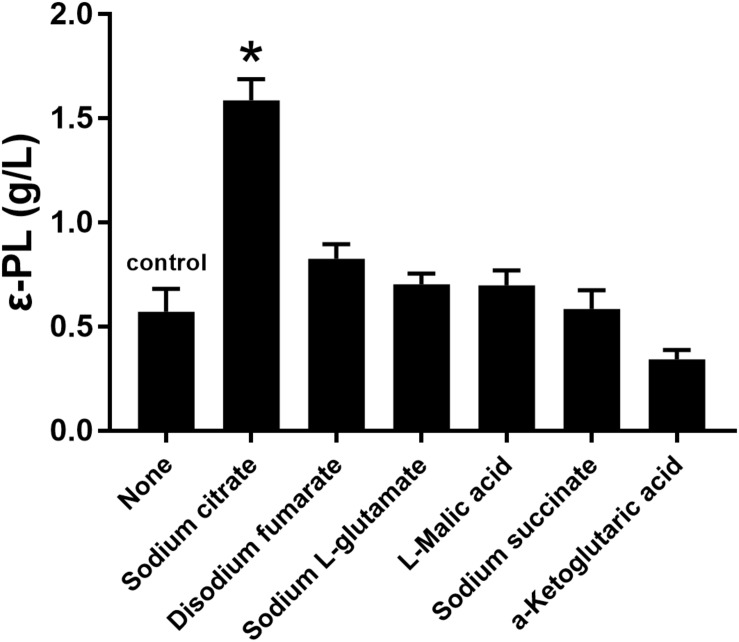
Effects of different metabolic intermediates on ε-PL production by the *pls* gene overexpressing strain Q-PL2. Data represent the means of three separate experiments, and error bars represent the standard deviation. Some error bars cannot be seen due to small standard deviations. *p < 0.05; one-way ANOVA with Dunnett’s multiple comparisons test.

Several studies have indicated that the addition of citrate is beneficial for ε-PL production (Bankar and Singhal, 2011; Xia et al., 2014). Therefore, the effects of sodium citrate and other metabolic intermediates (2 g/L) on the ε-PL production capacity of the *pls* gene overexpressing strain Q-PL2 were studied, and the results are shown in Figure 3. The titer of ε-PL was significantly improved by adding sodium citrate in M3G medium, while other metabolic intermediates had little effect on ε-PL production by strain Q-PL2.

**FIGURE 4:**
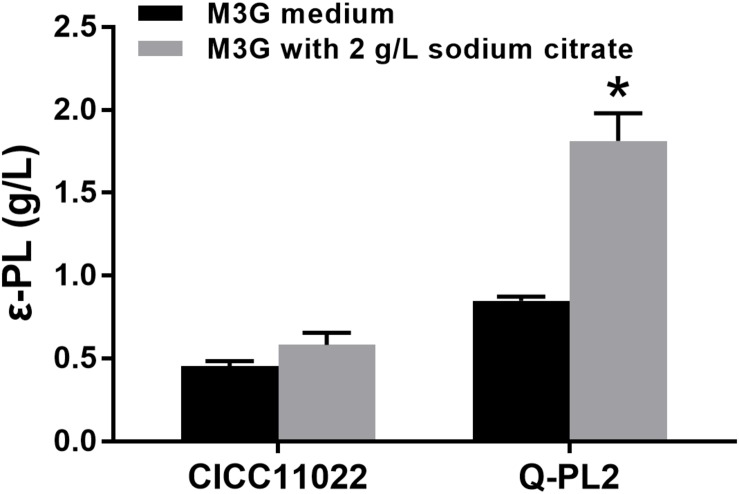
Synergistic effect of *pls* gene overexpression and citrate on ε-PL production. Data represent the means of three separate experiments, and error bars represent the standard deviation. Some error bars cannot be seen due to small standard deviations. *p < 0.05; two-way ANOVA with Sidak’s multiple comparisons test.

Then, the effects of sodium citrate on the ε-PL production abilities of the two strains were compared, and the results are shown in Figure 4. By adding 2 g/L sodium citrate, the ε-PL titers of *S. albulus* CICC 11022 increased from 0.45 ± 0.03 to 0.58 ± 0.07 g/L, while the ε-PL titers of strain Q-PL2 increased from 0.85 ± 0.02 to 1.81 ± 0.17 g/L. The increased ratios of ε-PL titers caused by sodium citrate to strain CICC 11022 and Q-PL2 were 28.5 ± 7.0 and 113.2 ± 13.3%, respectively. Hence, the addition of sodium citrate can synergize with the overexpression of the *pls* gene. With the synergistic effect of 2 g/L sodium citrate, the titers of ε-PL produced by Q-PL2 were 211.2% ± 17.4% higher than that produced by the wild strain.

### Shake Flask Fermentation of ε-PL

**FIGURE 5:**
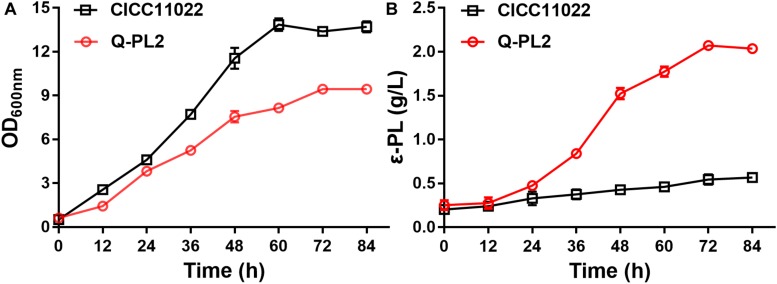
Cell growth and ε-PL production of *S. albulus* CICC 11022 and Q-PL2 under shake flask fermentations. **(A)** *S. albulus* CICC 11022. **(B)** *S. albulus* Q-PL2. Data represent the means of three separate experiments, and error bars represent the standard deviation. Some error bars cannot be seen due to small standard deviations.

To further confirm the synergistic effect of 2 g/L sodium citrate and *pls* gene overexpression, the cell growth and ε-PL production of the two strains at different time points were studied in shake flask fermentations. The maximum OD values of *S. albulus* CICC 11022 and Q-PL2 were 13.9 ± 0.4 and 9.5 ± 0.2, respectively (Figure 5A). The maximum ε-PL titers of *S. albulus* CICC 11022 and Q-PL2 were 0.57 ± 0.03 and 2.07 ± 0.1, respectively (Figure 5B). These results indicate that the synergistic effect of *pls* gene overexpression and sodium citrate can decrease cell growth and significantly increase the production of ε-PL.

### Optimization of the Fermentation Conditions

**FIGURE 6:**
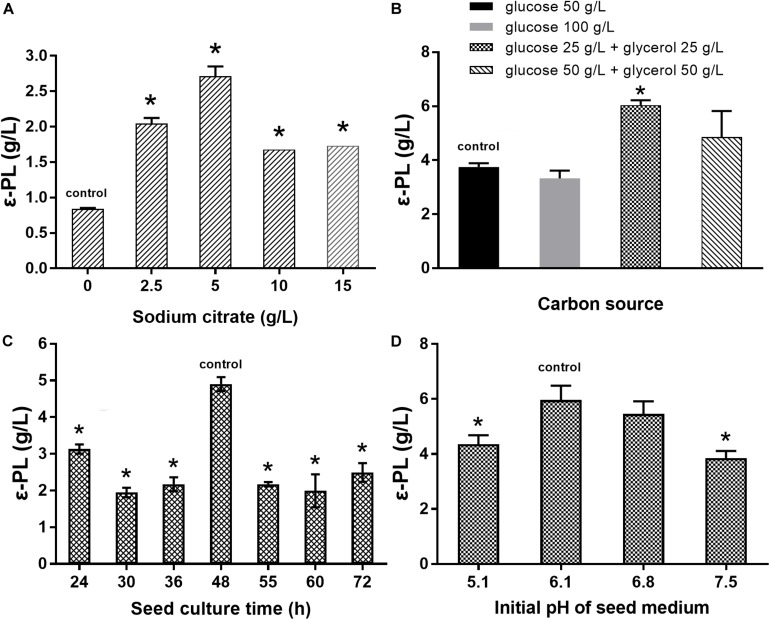
Optimization of the fermentation conditions for ε-PL production by *S. albulus* Q-PL2. **(A)** Effect of sodium citrate concentration on ε-PL production. **(B)** Effects of carbon source on ε-PL production. **(C)** Effects of seed culture time on ε-PL production. **(D)** Effects of initial pH of seed medium on ε-PL production. Data represent the means of three separate experiments, and error bars represent the standard deviation. Some error bars cannot be seen due to small standard deviations. *p < 0.05; one-way ANOVA with Dunnett’s multiple comparisons test.

The effects of sodium citrate concentration, carbon source, seed culture time, and initial pH of seed medium on ε-PL production were studied to obtain an optimal fermentation condition for *S. albulus* Q-PL2. As shown in Figure 6A, the best sodium citrate concentration was 5 g/L. Zeng et al. (2017) reported that the mixed carbon source of glucose and glycerol lead to higher ε-PL production, and this was verified in our study. The best carbon sources were 25 g/L glucose and 25 g/L glycerol (Figure 6B). In addition, the best seed culture time and initial pH of seed medium were 48 h and 6.1, respectively (Figures 6C,D). These optimal fermentation conditions were applied in fed-batch fermentations.

### Fed-Batch Production of ε-PL

**FIGURE 7:**
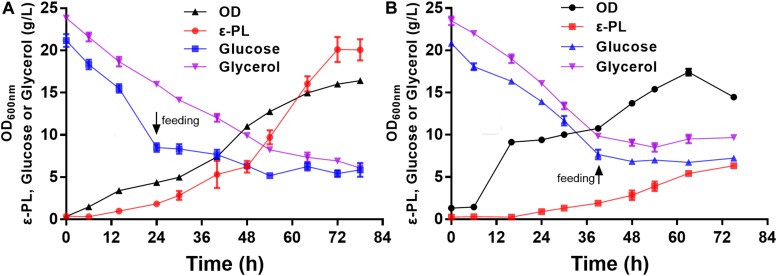
Fed-batch fermentations of *S. albulus* Q-PL2 and CICC 11022. **(A)** *S. albulus* Q-PL2. **(B)** *S. albulus* CICC 11022. Data represent the means of three samples per time interval, and error bars represent the standard deviation. Some error bars cannot be seen due to small standard deviations.

To further verify the ε-PL production ability of the *pls* gene overexpressing strain Q-PL2, fed-batch fermentations were performed, and the results are shown in Figure 7. During the entire fermentation process, the cell growth of the wild strain CICC 11022 was better than that of Q-PL2, but more ε-PL was produced by Q-PL2. The maximum specific growth rate, product formation rate, glucose and glycerol consumption rate of *S. albulus* Q-PL2 were 0.56 h^–1^, 0.63, 0.70, and 0.37 g/L/h, respectively. These values of *S. albulus* CICC 11022 were, in turn, 0.53 h^–1^, 0.18, 0.44, and 0.44 g/L/h, respectively. The ε-PL yields produced by strains Q-PL2 and CICC 11022 on carbon sources were 9.1 and 3.5% (mass ratio), respectively. After 72 h of fermentation, 20.1 ± 1.3 g/L of ε-PL was produced with a productivity of 6.7 ± 0.4 g/L/day by strain Q-PL2 (Figure 7A), whereas only 6.3 ± 0.4 g/L of ε-PL was produced with a productivity of 2.1 ± 0.1 g/L/day by strain CICC 11022 (Figure 7B). The titer and productivity of ε-PL produced by strain Q-PL2 was 3.2 ± 0.3-fold of that produced by strain CICC 11022.

## Discussion

Genetic engineering is one of the important methods to increase the fermentation ability of industrial production strains. But, up to now, fewer researches on enhancing ε-PL biosynthesis by genetic manipulation have been reported. Among these reports, most are focused on alleviating the restriction of environmental factors on fermentation, and very few studies have focused on the key enzymes in the ε-PL metabolic pathway. Dissolved oxygen is one of the key factors affecting ε-PL production. Xu et al. (2015) integrated *Vitreoscilla* hemoglobin gene (*vgb*) into the chromosome of *S. albulus* PD-1 to alleviate oxygen limitation during fermentation. Finally, the production of ε-PL was increased from 22.7 to 34.2 g/L with a productivity of 4.9 g/L/day. Gu et al. (2016) inserted the *vgb* gene and S-adenosylmethionine synthetase gene (*metK*) into the chromosome of *S. albulus* NK660 for expression, and the ε-PL titer increased from 0.6 to 0.76 g/L. Nitrogen source is another key factor restricting the synthesis of ε-PL. Each molecule of lysine contains two nitrogen atoms. Therefore, the synthesis of intracellular lysine requires more nitrogen sources. In order to solve this problem, the ammonium transporter gene (*amtB*) was overexpressed in *S. albulus* PD-1, and the production of ε-PL increased from 22.7 to 34.2 g/L with a productivity of 5.1 g/L/day (Xu et al., 2018). Aspartate kinase (Ask) is a key enzyme in the L-lysine synthetic pathway. Hamano et al. (2007) found that Ask of *S. albulus* NBRC14147 was partially regulated by feedback inhibition, so they constructed a mutant protein rAsk (M68V) that was completely unregulated by feedback inhibition and expressed the mutant protein rAsk in *S. albulus* CR1. As a result, the concentration of ε-PL increased from 12 to 15 g/L with a productivity of 2.1 g/L/day. In our research, the final concentration of ε-PL increased from 6.3 to 20.1 g/L by overexpressing *pls* gene in *S. albulus* and the synergistic effect of citrate. The productivity of ε-PL reached 6.7 g/L/day, which was much higher than other genetically engineered strains.

ε-PL synthase is the last enzyme in the ε-PL synthesis pathway. The function mechanism of this enzyme is well characterized (Yamanaka et al., 2008), but whether this enzyme is a key node that limits ε-PL synthesis remains unclear. Overexpression of Pls is expected to increase the production of ε-PL, but many attempts to use the constitutive promoter *ermE*^∗^ to overexpress Pls have failed (Yamanaka et al., 2011; Geng et al., 2014; Xu et al., 2019). In this study, an engineered strong promoter *kasO*p^∗^ (Wang et al., 2013) was used to achieve overexpression of the *pls* gene in *S. albulus* CICC 11022. During fermentation, the expression levels of the *pls* gene in the genetically engineered strain Q-PL2 were always 5-fold higher than those in the wild strain, but the final titer of ε-PL was only increased by 88.9%. These results indicate that the lack of intracellular L-lysine is likely to be a key factor limiting the production capacity of the *pls* gene overexpressing strain Q-PL2. We tried to add various metabolic intermediates to the fermentation medium and found that only the addition of citrate significantly increased the production of ε-PL by the *pls* gene overexpressing strain Q-PL2 (Figure 3). Xia et al. (2014) found that the addition of citric acid decreased the activities of pyruvate kinase, citrate synthase, and isocitrate dehydrogenase and increased the activity of aspartate aminotransferase in *S. albulus* PD-1. They deduced that citric acid feeding resulted in metabolic flux redistribution at the node of phosphoenolpyruvate. The metabolic pathway from phosphoenolpyruvate to tricarboxylic acid cycle was weakened and from phosphoenolpyruvate to oxaloacetate and L-aspartate was enhanced. ε-PL production was improved consequently because L-aspartate is the precursor of L-lysine synthesis (Xia et al., 2014). Additionally, tricarboxylic acid cycle is important for ATP generation because high levels of ATP are required for full enzymatic activity of Pls (Yamanaka et al., 2010). Therefore, we speculate that the addition of citrate can not only cause the accumulation of intracellular L-lysine, but also serves as a substrate to maintain the tricarboxylic acid cycle to produce sufficient ATP. However, even if the concentration of L-lysine and ATP is high enough, ε-PL cannot be produced sufficiently by the wild strain because the activity of Pls is not enough. This is probably the reason of the synergistic effect of *pls* gene overexpression and citrate on ε-PL production by *S. albulus* Q-PL2.

In summary, *pls* gene was overexpressed in *S. albulus* CICC 11022 by using a non-native promoter, and an enhanced ε-PL production was obtained with the synergistic effect of the gene and citrate. The results indicate that Pls is one of the rate-limiting enzymes in ε-PL synthesis pathway and lay a foundation for further improving the ε-PL production ability of *S. albulus* by metabolic engineering.

## Data Availability Statement

The datasets generated for this study are available on request to the corresponding author.

## Author Contributions

BY and JQ designed the research. AW and WT performed the experiments. XW, LC, and YX analyzed the data. XW and JQ wrote the manuscript. All authors read and approved the final manuscript.

## Conflict of Interest

The authors declare that the research was conducted in the absence of any commercial or financial relationships that could be construed as a potential conflict of interest.
