# Supplementary material for: Enhanced ε-Poly-L-Lysine Production by the Synergistic Effect of ε-Poly-L-Lysine Synthetase Overexpression and Citrate in Streptomyces albulus
Source: Front Bioeng Biotechnol. 2020 Apr 22;8:288. doi: 10.3389/fbioe.2020.00288 (PMC7188835; doi:10.3389/fbioe.2020.00288)
Supplement: Supplementary file 1 [file Data_Sheet_1.docx]

**Supplementary Material**

**Enhanced ε-poly-L-lysine production by the synergistic effect of ε-poly-L-lysine synthetase overexpression and citrate in *Streptomyces albulus***

**Aixia Wang^1^, Wenzhe Tian^1^, Lei Cheng^2^, Youqiang Xu^2^, Xiuwen Wang^1*^, Jiayang Qin^1*^, Bo Yu^3^**

^1^College of Pharmacy, Binzhou Medical University, Yantai 264003, PR China

^2^Beijing Engineering and Technology Research Center of Food Additives, Beijing Technology & Business University (BTBU), Beijing 100048, PR China

^3^CAS Key Laboratory of Microbial Physiological and Metabolic Engineering, Institute of Microbiology, Chinese Academy of Sciences, Beijing 100101, PR China

*Corresponding author

Email addresses:

AW: [406076940@qq.com](mailto:406076940@qq.com); WT: [1158807207@qq.com](mailto:1158807207@qq.com);

LC: [chenglei@btbu.edu.cn](mailto:chenglei@btbu.edu.cn); YX: [xuyouqiang@btbu.edu.cn](mailto:xuyouqiang@btbu.edu.cn);

XW: [wangxiuwen0822@163.com](mailto:wangxiuwen0822@163.com);

JQ: [qinjysdu@163.com](mailto:qinjysdu@163.com); BY: [yub@im.ac.cn](mailto:yub@im.ac.cn)

**Table S1.** Oligonucleotides used to construct overexpression plasmids.

| Oligonucleotides | Sequence |
| --- | --- |
| pro1 | gccaagcttgggctgcaggtcgactctagatgttcacattcgaacggtctctgctttg |
| pro2 | tgttcacattcgaacggtctctgctttgacaacatgctgtgcggtgttgtaaagtcgtggccaggagaata |
| pro4 | atggacactccttacttagatctagtattctcctggccacgactttacaacaccgcacagcatgtt |
| pls-F2 | tactagatctaagtaaggagtgtccatatgtcgtcgccccttctcgaatcgtccttc^2^ |
| pls-R | caggaaacagctatgacatgattacgaattctcacgcggccgcacctccctccgcgcg |

^1^The underlined sequence is RBS2.

**Table S2.** Primers for qRT-PCR analysis with target gene information

| **Gene ID** | **Description** | **Primers (5′→3′)** | **Length** |
| --- | --- | --- | --- |
| SAZ_36495 | ε-Poly-L-lysine synthase | GCGAGATGTGGAACACCTACGG  GCGAGCTGCCAGCCCTTCA | 115 bp |
| SAZ_12395 | RNA polymerase sigma factor (hrdB) | CTGACCAGATTCCGCCAACCC  GCCTCTGCGGCACTGACCAT | 100 bp |

**Fig. S1.** Diagram of the integrative plasmids containing *pls* gene, strong promoter, and RBS2.


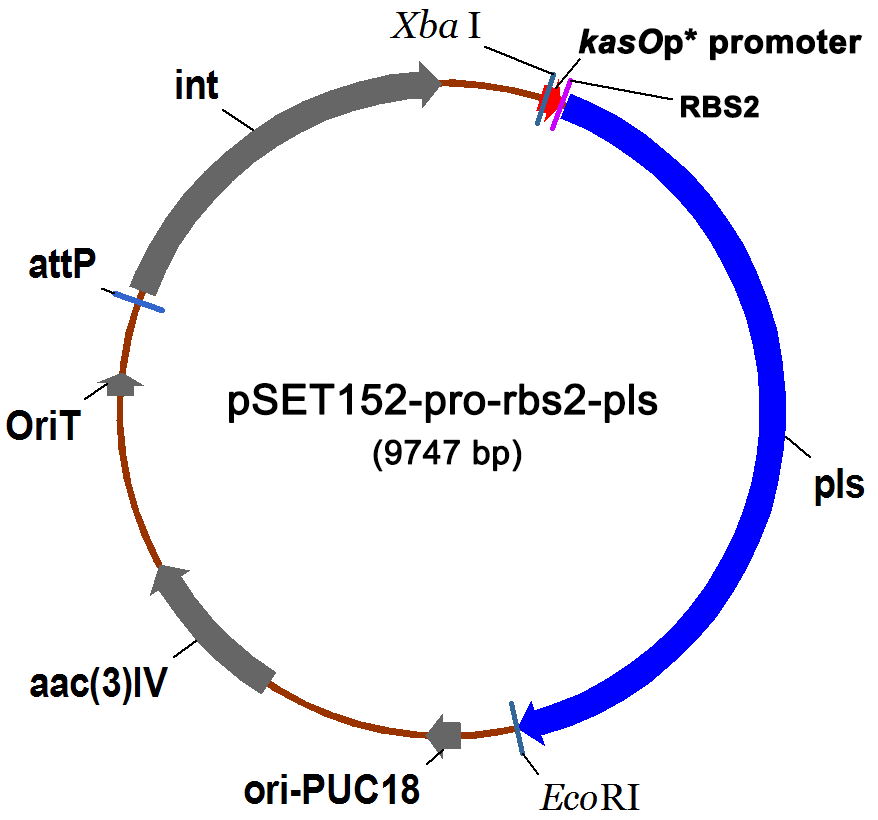


**Fig. S2.** The description and assembled sequences used in this study.

Sequence of *pro-rbs2-pls*

(5′ flank-XbaI-*kasO*p* promoter-RBS2-*pls* gene-EcoRI-3′ flank)

gccaagcttgggctgcaggtcgactctagatgttcacattcgaacggtctctgctttgacaacatgctgtgcggtgttgtaaagtcgtggccaggagaatactagatctaagtaaggagtgtccatatgtcgtcgccccttctcgaatcgtccttcgagccgtccgagccagcgccccaacaggccctgtaccgcaccgccggcaacccggccccgcggaccctgctcgacgtgctcgatgccaccgccgccgcacatccccaggcgatcgccctggacacgggctccgaggcgctcacctaccgcgacctgtgtatcgagatcgaacgccgcgcacggcagctcagggaccgcggcatcggtcccggcgaccgggtcggagtccgcgtcccctccgggaccgccgagctgtacctgtccatcctcgccgtcctgcgcagcggagcggcctacgtgccggtcgacgccgacgaccccgacgagcgggccgccaccgtcttccgcgaggccgccgtctgcgccgtcctcggccccgacggcccgctgcccggcccggcccggcccctcggcgacccgcgttccgcgggcccccaggacgacgcctggatcatcttcacctcgggttcgaccggcgcgcccaagggcgtggcggtcagccaccgctccgccgccgccttcgtcgacgccgaggccgacctgttctgccaggaccagccgttgggccccggcgaccgggtgctggccgggctgtccgtcgccttcgacgcctcctgcgaggagatgtggctcgcctggcggtacggcgcctgcctggtgcccgcaccccgcgcgctggtccgggccggccacgaactcggcccctggctcgtcgagcgcggcatcaccgtcgtctccaccgtgcccaccctcgccgcgctctggccggacgaggcgatgcgccgggtccgcctgctgatcgtcggcggcgaatcctgcccggccgggctcgtcgaccgcttcgccggacccggccgcgagatgtggaacacctacggcccgaccgagaccaccgtcgtcgcctgcgccgcccgcctgctgccgggcgagccggtccgcatcggcctgcccctgaagggctggcagctcgccgtcgtcgaccgcaccgggcagccggtgcccttcggcgccgagggcgaactgctgatcagcggcgtcggcacggcccgctacctcgaccccgccaaggacgccgaacggttccggcccgacgacgccctgggggccgcccgcgtctaccgcaccggcgacctggtccgggccgaacccgagggcctgctcttcgtcggccgcgccgacgaccagatcaaactcggcggccgccgcatcgagctgggcgagatcgacgccgccctggccgccctgcccggcgtccgcggggccgccgcggccgtccagacgacgccggccggcacccaggtgctggtcggctacgtcgttcccgagcagcgcaccgccgacggttccagcttccagcaggacaaggcccgcgcactgctccaggaacgcctgcccgcgcagttggtcccggtcctcgcggaggtcgagtccctgcccacccggacctccggcaaggtcgaccgcaaggcgctgccctggccgctgccgtccgccccggtcgactccgccaccggcgatccggccacggcgctggacggcaccgccgcccggctcgccgggatctgggaggaactcctcggcgtccggcccggcccggacagcgacttcgtctccctcggcggcaccagcctggtcgccgcccgcatggcgtcccagctccgcatccaccaccccggcgtctcggtcgccgacctctaccgccacccggtgctgcgcgacatggccgagcacctcgactcgctgggcggcccggtggacgaggtccgcccggtccgccccgtcccgcgccgcaccggattcgtccaactcctcgtccagaccggcctgtacggcatcgccggcctgcgcggactggtcgggctcgcgctcgcggacaacgtcctcggcctgctcgccccgcaggtctgggccccgcacaccgcgtggtggctgatcatcgtcggctgggtggtgctctacagcgccccgatgcgttgcgccctcggcgcactggccgcccgcgcgctcgccggcaccatcaagcccggcgcctacccgcgcggcggcgccacccacctgcgcctgtggaccgccgaacgcgtcgtcgccgccttcggcgtcccctccctgctcggcaccccctgggcgcggctctacgcccggagcctgggctgcgccacagggcggaacgtggcgctgcacaccatgccgccggtcaccggcctcgccgaactcggcgacggctgcagcgtcgaacccgaggccgacatctccggctggtggctcgacggcgacaccctgcacatcggcgcggtccggatcggcgccggcgcccgggtcgcccaccgcagcatgctgatgcccggcgccgtcgtcggccagggcgccgaactcgcctccggcgcctgcctggacggagagatccccgacggcgcctcgtggtccggctccccggcccgcccggccggcgccgccgagcggatggccggcgccgcctggcccgcccccgcctggcagcgctcgcgccgctggagcgccgcctacggactgaccctgctgggcctgccgctgctggccctgctgtccaccgcgcccgccctggtcggcgcgtacttcctgctccgcgacagcggcaccctcgccacagccgggcttcgcctgctgctggccgtcccggtcttcacgctcctgaccactggctgctccctcctcgtcaccgccgccgtggtgcgcctcctcggccgcggcatcacgccgggactgcaccccgcgagcggtggcgtcgcctggcgcgcctggctggtcacccgcctcctggacggcgcccgcggcagcctcttcccgctctacgccagcctcggcaccccgcactggctgcggctgctcggcgccaaggtcggccggcacgcggagatctccaccgtgctgccgctgccctccctgctgcacgtcgaggacggcgcgttcctcgccgacgacaccctggtggcgcccttcgaactccgcggcggctggctgcggttggggaccgtccggatcggtcgccgggccttcgtcggcaactccggcatcgtcgaccccggccacgacgtgcccgatcacagcctggtcggcgtgctctccaacgcccccgccgacggcgagcccggctcgtcctggctgggccggcccgccatgccgctgccccgggtggcgacccaggccgacccggcgcgcaccttcgcaccgccgcgcaggctggtccgggcccgcgccgccgtcgagctgtgccgggtgctgccgctgatgtgcggcctggcgctcgccgagggcgtgttcctcaccgagcaggacgccttcgcccagggcggcctcggtctcgccgcactggtcggcgccccgctgctgctggcctcgggcctcgtggcgctgctcgtcaccaccctcgcgaagtggctgctggtcggccgcttcacggtgagcgagcaccccctgtggtcgtcgttcgtgtggcgcaacgagctctacgacaccttcgtcgaatcgctcgccgtgccgtcgatggccggcgcgttcaccggcaccccggtcctgaactggtggctgcgcaccctcggcgccaagatcgggcgcggggtctggttggagagctactggctgccggagaccgacctgatcaccgtcgccgacggcgtcagcgtcaaccgcggctgcgtcctgcagacccacctcttccacgaccggatcatgcggctggacaccgtccgcctcgccgaaggctcctcgctcggcccgcacggcatcgtgctccccggcaccgaggtcggggcgcgcgcctcgatcgcgccgtcgtccctggtcatgcgcggcgagagcgtcccggcccacacccggtgggccggcaacccgatcgccggcgaacgccccgcccgccccgtcccggcacgcgcggagggaggtgcggccgcgtgagaattcgtaatcatgtcatagctgtttcctg
